# Using Natural Language Processing to Predict Fatal Drug Overdose From Autopsy Narrative Text: Algorithm Development and Validation Study

**DOI:** 10.2196/45246

**Published:** 2023-05-19

**Authors:** Leigh Anne Tang, Jessica Korona-Bailey, Dimitrios Zaras, Allison Roberts, Sutapa Mukhopadhyay, Stephen Espy, Colin G Walsh

**Affiliations:** 1 Department of Biomedical Informatics Vanderbilt University Medical Center Nashville, TN United States; 2 Office of Informatics and Analytics Tennessee Department of Health Nashville, TN United States

**Keywords:** fatal drug overdose, natural language processing, surveillance, Tennessee, State Unintentional Drug Overdose Reporting System, SUDORS

## Abstract

**Background:**

Fatal drug overdose surveillance informs prevention but is often delayed because of autopsy report processing and death certificate coding. Autopsy reports contain narrative text describing scene evidence and medical history (similar to preliminary death scene investigation reports) and may serve as early data sources for identifying fatal drug overdoses. To facilitate timely fatal overdose reporting, natural language processing was applied to narrative texts from autopsies.

**Objective:**

This study aimed to develop a natural language processing–based model that predicts the likelihood that an autopsy report narrative describes an accidental or undetermined fatal drug overdose.

**Methods:**

Autopsy reports of all manners of death (2019-2021) were obtained from the Tennessee Office of the State Chief Medical Examiner. The text was extracted from autopsy reports (PDFs) using optical character recognition. Three common narrative text sections were identified, concatenated, and preprocessed (bag-of-words) using term frequency–inverse document frequency scoring. Logistic regression, support vector machine (SVM), random forest, and gradient boosted tree classifiers were developed and validated. Models were trained and calibrated using autopsies from 2019 to 2020 and tested using those from 2021. Model discrimination was evaluated using the area under the receiver operating characteristic, precision, recall, *F*_1_-score, and *F*_2_-score (prioritizes recall over precision). Calibration was performed using logistic regression (Platt scaling) and evaluated using the Spiegelhalter *z* test. Shapley additive explanations values were generated for models compatible with this method. In a post hoc subgroup analysis of the random forest classifier, model discrimination was evaluated by forensic center, race, age, sex, and education level.

**Results:**

A total of 17,342 autopsies (n=5934, 34.22% cases) were used for model development and validation. The training set included 10,215 autopsies (n=3342, 32.72% cases), the calibration set included 538 autopsies (n=183, 34.01% cases), and the test set included 6589 autopsies (n=2409, 36.56% cases). The vocabulary set contained 4002 terms. All models showed excellent performance (area under the receiver operating characteristic ≥0.95, precision ≥0.94, recall ≥0.92, *F*_1_-score ≥0.94, and *F*_2_-score ≥0.92). The SVM and random forest classifiers achieved the highest *F*_2_-scores (0.948 and 0.947, respectively). The logistic regression and random forest were calibrated (*P*=.95 and *P*=.85, respectively), whereas the SVM and gradient boosted tree classifiers were miscalibrated (*P*=.03 and *P*<.001, respectively). “Fentanyl” and “accident” had the highest Shapley additive explanations values. Post hoc subgroup analyses revealed lower *F*_2_-scores for autopsies from forensic centers D and E. Lower *F*_2_-score were observed for the American Indian, Asian, ≤14 years, and ≥65 years subgroups, but larger sample sizes are needed to validate these findings.

**Conclusions:**

The random forest classifier may be suitable for identifying potential accidental and undetermined fatal overdose autopsies. Further validation studies should be conducted to ensure early detection of accidental and undetermined fatal drug overdoses across all subgroups.

## Introduction

### Background

In recent years, incidences of fatal drug overdose have surged in the United States. During the COVID-19 pandemic, the per capita monthly overdose death rate increased by 60% in May 2020 compared with May 2019 [1]. At the state level, Tennessee recorded an increase of 98% from May 2019 to May 2020 and was surpassed by only West Virginia and Kentucky. Since the pandemic, the rate of increase in fatal overdoses has slowed; however, fatal drug overdoses remain a public health concern with a substantial economic burden [2,3]. Provisional national data estimated that 107,622 drug overdose deaths occurred in 2021, an increase of 15% from 2020 [2]. Meanwhile, Tennessee has continued to surpass national trends, recording 3814 overdose deaths in 2021, a 26% increase from 2020 [4]. This stark increase in overdose deaths calls for improved surveillance, intervention, and prevention work to confront the overdose crisis at the state level.

Fatal drug overdose surveillance provides critical information for prevention and intervention. Specifically, surveillance helps identify where fatal overdoses occur, what substances were involved, and whether those trends have changed over time, all of which help make response efforts more targeted and relevant [5]. Data timeliness enables more effective and immediate intervention. The Tennessee Department of Health (TDH) currently conducts fatal drug overdose surveillance through the State Unintentional Drug Overdose Reporting System (SUDORS). SUDORS captures the details associated with accidental and undetermined fatal overdoses using death certificates, death scene investigations, autopsies, toxicology reports, and prescription drug monitoring program data. SUDORS is nested within the National Violent Death Reporting System and is funded by the Overdose Data to Action grant from the Centers for Disease Control and Prevention [6].

Although SUDORS provides a wealth of knowledge pertaining to fatal drug overdoses, a delay of 6 months occurs from the time of death to data collection. The delay is needed for death certificate coding to be completed and autopsy reports to become available for abstraction [7,8]. Notably, recent efforts to facilitate rapid fatal drug overdose surveillance have leveraged natural language processing (NLP). Several studies have explored the application of NLP to social media data for more timely fatal drug overdose surveillance [9-11]. Although potentially available closer to real time, social media data are subject to other challenges such as selection bias (social media users vs nonusers), user privacy settings limiting access to posts, and observer effects altering user behaviors [12]. Other studies have applied NLP to more traditional data sources for surveillance. Using death certificates, some surveillance teams have identified drug overdose deaths from cause of death fields available before the final cause of death coding [13-15]. Other teams have used text from verbal autopsies, which describe interviews with witnesses or relatives, as opposed to forensic autopsies, which detail findings from extensive physical examinations and toxicology tests [16,17]. Text from forensic autopsy reports has been used for the automatic classification of causes of death unrelated to drug overdoses [18]. However, to the best of our knowledge, no studies have leveraged free text in forensic autopsy reports to specifically predict fatal overdoses. Autopsy narrative text is similar to scene evidence descriptions in medicolegal documents, which are available sooner than autopsies.

### Objectives

Faster identification of fatal overdoses can facilitate timely prevention and response efforts. In partnership with a research team in Biomedical Informatics at Vanderbilt University Medical Center (VUMC), the TDH sought to use NLP to identify fatal drug overdose deaths using narrative text from forensic autopsy reports.

## Methods

### Tennessee Fatal Drug Overdose Data Abstraction Process

TDH operates within a decentralized medicolegal death investigation system. Each of the 95 counties reports to 1 of the 5 forensic centers, which send reports to the Tennessee Office of the State Chief Medical Examiner (OSCME). The OSCME manages a repository of autopsy reports from all the forensic centers. Using the International Classification of Diseases, Tenth Revision (ICD-10) codes and keywords in death certificates, the SUDORS team identifies potential fatal overdoses. The SUDORS team obtains access to relevant autopsy reports and manually reviews each report to abstract variables into a REDCap (Research Electronic Data Capture) database (hereafter referred to as the SUDORS database) [19,20]. The SUDORS database contains information such as basic demographics, location of death, cause of death, and death scene information. As part of routine work at the TDH, at least 2 SUDORS abstractors review each case to ensure accurate coding.

### Data Sources

NLP models were developed using text from forensic autopsy reports describing all manners of death from 2019 to 2021. Autopsies included all fatal overdose deaths identified by the SUDORS team (cases) and the remaining other-cause deaths (controls) in the OSCME repository from 2019 to 2021, as of August 31, 2022. Autopsies were in semistructured forms containing common headings (detailed below) and computer-entered text. Autopsies were received as faxes or scans (PDFs) and processed using Adobe Acrobat Pro optical character recognition (OCR; Adobe). The outcome of interest was whether a given autopsy described an accidental or undetermined (ie, could not be definitively declared as accidental) fatal drug overdose, as indicated by the SUDORS team in the SUDORS database.

Narrative sections were defined as free-text portions of the autopsy that included the death scene information and medical history. These sections were commonly preceded by headings such as “summary of case,” “summary and interpretation,” “summary and opinion,” and “narrative summary.” The SUDORS team deemed these narrative sections to be the most informative parts of the autopsy report for determining the cause of death. Other data elements in the autopsy report varied substantially in quality (scanned toxicology results from different laboratories) or contained information that was interpreted and summarized in narrative sections (internal examination findings, external examination findings, and toxicology results). As such, this approach was limited to narrative sections to improve generalizability to other data sources in the future, such as medical examiner scene investigation notes, which are similar in content to the autopsy narrative text. This study was conducted before medical examiner notes were made available to the SUDORS team.

### Preprocessing of Narrative Text

Autopsy PDFs were converted to a binary format and parsed page by page into text. Symbols that were likely artifacts of the OCR process (as determined by a manual review) were removed using regular expressions. The autopsy report templates were semistructured and unique to each forensic center. Template text patterns were identified by manual review and used to extract the forensic center issuing the autopsy, as well as relevant narrative sections. Autopsies for which a forensic center could not be identified using this templated language were removed from the data set.

To facilitate the comparison of information across report formats, narrative text was divided into three sections based on location within the autopsy report: (1) an initial narrative (beginning of report), (2) a case summary (middle of report and after internal and external examination findings), and (3) a summary of circumstances (end of report). Narrative sections within the same autopsy report were concatenated such that each report had only one combined narrative section.

**Figure 1:**
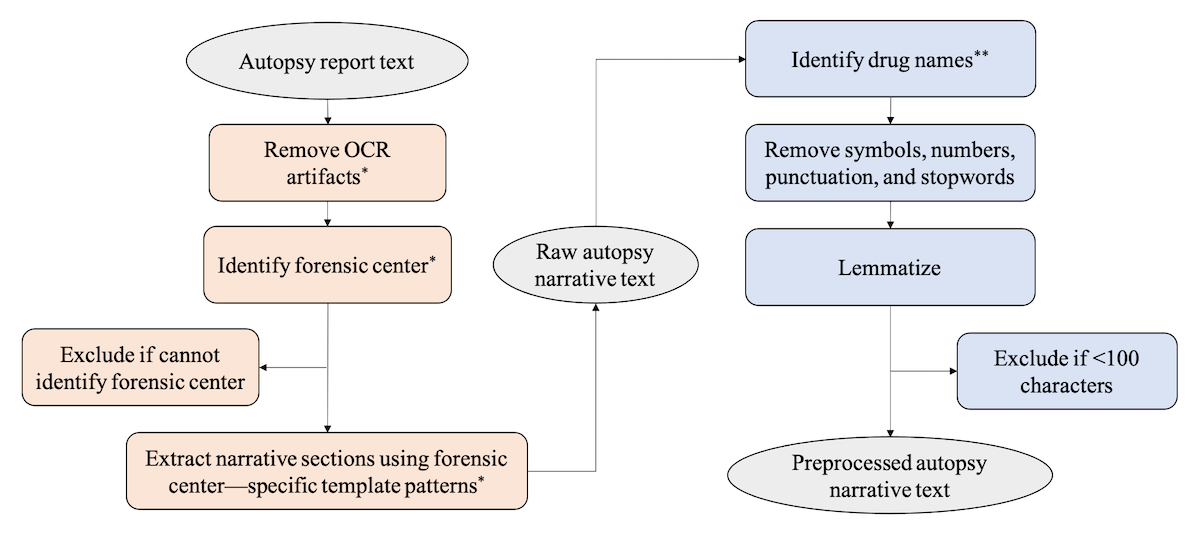
Autopsy narrative text extraction (orange) and preprocessing (blue) steps. *Regular expression patterns determined by manual review with input from SUDORS team. **Words with a number, hyphen, and letter were combined (eg, “4-ANPP” or 4-anilino-N-phenethylpiperidine converted to “4ANPP”). An exception was if the string contained “old” or “year” (eg, “35-year-old”). OCR: optical character recognition; SUDORS: State Unintentional Drug Overdose Reporting System.

The autopsy narrative text was extracted and preprocessed according to the steps outlined in Figure 1. A set of rules were developed using patterns found by manual review and enacted to remove additional artifacts resulting from the OCR process while ensuring the detection of drug names containing hyphens (eg, “4-ANPP” or 4-anilino-N-phenethylpiperidine) as one word rather than separate words (“4” and “ANPP”). Generalized regular expressions were used to identify potential references to drugs to account for novel drug combinations and varying OCR qualities. Words containing a number, hyphen, and letter were identified as potential drugs and combined (“4ANPP”). An exception to this rule was if the string contained “old” or “year,” in which case the string likely referred to age rather than a drug (“35-year-old”). The numbers were removed if they were detected in words that were not identified as potential drug names. Punctuation and stop words (with the exception of “none” and “nowhere”) were removed, and terms were lemmatized. Reports with fewer than 100 characters were likely not sufficiently processed via OCR and were thus removed from the data set.

### Modeling Plan

Autopsies from 2019 to 2020 were used for training (95%) and calibration (5%), and autopsies from 2021 were used for testing. This allowed model development to occur while the SUDORS team abstracted information from 2021 autopsies. Bag-of-words models were developed using term frequency–inverse document frequency scoring, which normalizes the word count in a given document based on the prevalence of the word among all documents, such that rarer words are given higher scores. Terms appearing in fewer than 20 documents in the training set or with fewer than 4 characters were excluded.

Logistic regression, support vector machine (SVM), random forest, and gradient boosted tree classifiers were generated. Hyperparameters for SVM, random forest, and gradient boosted trees were tuned with 5-fold cross-validation.

### Model Metrics

The area under the receiver operating characteristic (AUROC), precision, recall, *F*_1_-score (equal weighting of precision and recall), and *F*_2_-score (recall weighted heavier than precision) were calculated for the corresponding hold-out data sets. Model error was evaluated using 100 bootstrapped samples, and 95% CIs were generated for each metric. Recall was identified as the most important metric that ensured that all potential cases of fatal overdoses would be reviewed by the surveillance team. Therefore, the *F*_2_-score rather than *F*_1_-score was primarily used to compare model performance.

The SVM, random forest, and gradient boosted tree classifiers achieving the highest *F*_2_-scores were calibrated using Platt scaling, which applies logistic regression to normalize the predicted probabilities from the test set to the expected distribution of probabilities, as determined by the calibration set. The divergence between the predicted and expected probabilities of fatal overdoses was evaluated using the Spiegelhalter *z* test statistic. SHAP (Shapley additive explanations) values were generated for the random forest and gradient boosted tree classifiers [21].

### Post Hoc Subgroup Analysis

The random forest classifier, which achieved the highest *F*_2_-score and was calibrated, was used for post hoc subgroup analyses that assessed model performance within the test data set by forensic center, race, age at death, sex, and education level. Individuals in these subgroups were suspected to have varying levels of narrative text available for model prediction [22]. This analysis was also completed using the logistic regression model to ensure that the subgroup performances of the parametric (logistic regression) and nonparametric (random forest classifier) models were evaluated.

Subgroup information was extracted from the SUDORS database and death certificates (for non-SUDORS deaths or controls). In death certificates, race categories were mutually exclusive (ie, a decedent could only be coded as 1 race), whereas in the SUDORS database, more than 1 race could be indicated. To harmonize these data, race was coded as American Indian, Asian, Black, Other, Pacific Islander, or White. Individuals of multiple races were recoded as the most prevalent race within the population. For example, an individual coded as White (prevalence=0.73) and American Indian (prevalence=0.001) was recoded to White to reflect the more prevalent race in the data set. Age at death was recoded into ranges used by the National Center for Health Statistics: ≤14, 15-24, 25-34, 35-44, 45-54, 55-64, and ≥65 years [23]. The sex categories were female, male, and unknown. Education level was coded as 8th grade or less, 9th to 12th grade but no diploma, high school graduate or General Educational Development test, college but no degree, associate’s degree, bachelor’s degree, master’s degree, doctorate or professional degree, or unknown. Within each subgroup, AUROC, precision, recall, *F*_2_-score, cases, controls, and median narrative text length were calculated.

### Experimental Setup

This study was conducted in Python (version 3.9.7; Python Software Foundation) and R (version 4.2.0; R Foundation for Statistical Computing). Autopsy PDFs were converted to text using the *extract_text* function in the Python *pdfminer* package (version 20191125). Data preprocessing was performed using the *spaCy* package (version 3.2.4) and the *scispaCy* (version 0.5.0) *en_core_sci_sm* NLP pipeline, which was pretrained on biomedical data and had a vocabulary size of approximately 100K [24]. The models were constructed in Python using *scikit-learn* (version 1.1.1) and *xgboost* (version 1.6.1). The Spiegelhalter *z* test statistic was calculated using *rms* (version 6.3.0) in R, and the SHAP values were generated using SHAP (version 0.41.0) in Python.

### Ethical Considerations

This study was a quality improvement project found exempt by the TDH institutional review board.

## Results

### Overview

An initial set of 17,521 autopsies was obtained for all manners of death from 2019 to 2021. A subset of autopsies (179/17,521, 1%) was removed because of unsuccessful identification of a specific forensic center or insufficient characters (<100 characters) after text preprocessing, including lemmatization. The final data set contained 17,342 autopsies (5912 cases and 11,430 controls). The most common manners of death were *accidental* among cases (5811/5912, 98%) and *natural* among controls (3994/11,430, 35%; Multimedia Appendix 1). Forensic centers were deidentified and denoted as A, B, C, D, and E. Approximately half (8743/17,342, 50.42%) of the total autopsies were obtained from the forensic center A (Table 1). The proportion of fatal overdoses was the highest among autopsy reports from the forensic center C (1229/3007, 40.87%).

**Table 1:** Cases and controls by forensic center (columns), entire data set.

	Forensic center, n (%)						Total (N=17,342), n (%)
	A (n=8743)	B (n=3717)	C (n=3007)	D (n=1452)	E (n=423)		
Cases	3095 (35.39)	1015 (27.3)	1229 (40.87)	417 (28.72)	156 (36.87)	5912 (34.09)	
Controls	5648 (65.6)	2702 (72.69)	1778 (59.13)	1035 (71.28)	267 (63.12)	11,430 (65.91)	

### Narrative Text Sections

Across forensic centers, narrative text sections remained similar in content (each contained scene evidence and medical history) but had varying levels of missingness (Table 2). Notably, forensic centers A and B shared very similar formats, consisting of an initial narrative and a case summary. Autopsies from forensic centers C and D tended to have an initial narrative and summary of circumstances. Autopsies from forensic center E only presented a summary of circumstances.

**Table 2:** Sections of narrative text by forensic center in entire data set.

Forensic center	Initial narrative, n (%)	Case summary, n (%)	Summary of circumstances, n (%)	Total characters, median (IQR)
A (n=8743)	8675 (99.22)	8732 (99.87)	3 (0.03)	1287 (1053-1560)
B (n=3717)	3717 (100)	3687 (99.19)	0 (0)	1319 (1119-1566)
C (n=3007)	3007 (100)	0 (0)	2238 (74.43)	2852 (1855-4142)
D (n=1452)	1452 (100)	0 (0)	562 (38.71)	1074 (666-1511)
E (n=423)	0 (0)	0 (0)	423 (100)	462 (273-617)

### Model Development

Of the 17,342 autopsies, a subset of 10,215 autopsies was used for training (3342/10,215, 32.72% cases), 538 for calibration (183/538, 34.01% cases), and 6589 for testing (2409/6589, 36.56% cases). A total of 4002 terms from the training data comprised the vocabulary set for the bag-of-words models.

### Model Validation

All models achieved AUROC≥0.95, precision≥0.94, recall≥0.92, *F*_1_-score ≥0.94, and *F*_2_-score ≥0.92 (Table 3). The highest recall and *F*_2_-scores were achieved using the SVM (recall=0.948, 95% CI 0.937-0.959; *F*_2_-score =0.948, 95% CI 0.94-0.957) and random forest (recall=0.942, 95% CI 0.933-0.954; *F*_2_-score =0.947, 95% CI 0.939-0.956). The corresponding 95% CIs for these metrics did not overlap with those for logistic regression, suggesting that SVM and random forest achieved significantly higher recall and *F*_2_-scores than logistic regression (*P*<.05). The logistic regression and random forest were calibrated (*P*=.95 and *P*=.85, respectively). The SVM and gradient boosted tree classifiers were miscalibrated (*P*=.03 and *P*<.001, respectively).

**Figure 2:**
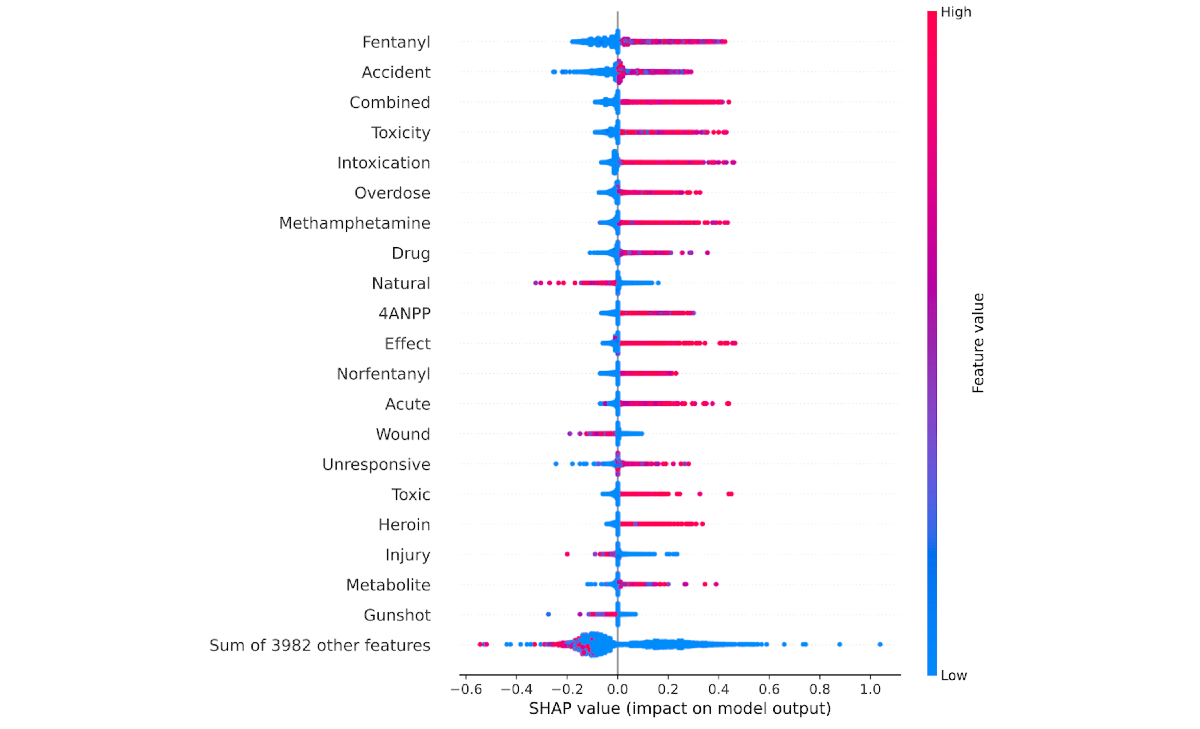
Top 20 SHAP (Shapley additive explanations) values for the random forest classifier. 4ANPP: 4-anilino-N-phenethylpiperidine.

The SHAP value analysis indicated that “fentanyl,” “accident,” “toxicity,” and “combined” were among the top features for the random forest (Figure 2) and gradient boosted trees classifiers (Multimedia Appendix 2). “Natural” was most strongly associated with controls.

**Table 3:** Model discrimination and classification metrics across 100 bootstrapped samples.

	Area under the receiver operating characteristic, median (95% CI)	Precision, median (95% CI)	Recall, median (95% CI)	*F*_1_-score, median (95% CI)	*F*_2_-score^a^, median (95% CI)	*P* value^b^
Logistic regression	0.95 (0.943-0.955)	0.963 (0.954-0.972)	0.92 (0.908-0.928)	0.941 (0.933-0.946)	0.928 (0.918-0.935)	−0.057 (.95)
Support vector machine	0.96 (0.954-0.966)	0.95 (0.936-0.962)	0.948 (0.937-0.959)	0.949 (0.942-0.956)	0.948 (0.94-0.957)	2.17 (.03)
Random forest	0.961 (0.955-0.967)	0.964 (0.954-0.973)	0.942 (0.933-0.954)	0.954 (0.945-0.96)	0.947 (0.939-0.956)	−0.189 (.85)
Gradient boosted trees	0.954 (0.946-0.962)	0.949 (0.935-0.964)	0.937 (0.92-0.953)	0.943 (0.935-0.953)	0.939 (0.926-0.951)	−6.79 (<.001)

^a^*F*_2_-score prioritizes maximizing recall over precision.

^b^Spiegelhalter *z* test statistic.

### Subgroup Analysis

The subgroup analysis revealed similar results for the random forest classifier (Table 4) and logistic regression model (Multimedia Appendix 3), with a few exceptions. Across forensic centers for both models, forensic centers A, B, and C had the highest *F*_2_-scores, whereas forensic centers D and E had the lowest *F*_2_-scores. Across race categories for random forest, *F*_2_-score was highest for Pacific Islander (*F*_2_-score =1) and lowest for American Indian (*F*_2_-score =0.833) and Asian (*F*_2_-score =0.862). The logistic regression model achieved similar metrics but performed better for the American Indian race category (*F*_2_-score =1). Across age groups for the random forest, *F*_2_-score was highest for age groups 15-24 years (*F*_2_-score =0.973) and lowest for ≤14 years (*F*_2_-score =0.6) and ≥65 years (*F*_2_-score =0.789). The same relative order of *F*_2_-scores was preserved for logistic regression, but performance was considerably worse for age group ≤14 years (*F*_2_-score =0.227). Models performed similarly across sex and education levels. Notably, several subgroups included fewer than 20 cases or controls.

**Table 4:** Random forest discrimination and classification metrics, cases, controls, and narrative section length for each subgroup in the test set (n=6589).

Subgroup		Area under the receiver operating characteristic	Precision	Recall	*F*_2_-score	Cases, n (%)	Controls, n (%)	Length, median (IQR)
**Forensic center**								
	A	0.978	0.971	0.973	0.973	1192 (18.09)	2038 (30.93)	1256 (1040-1497)
	B	0.975	0.966	0.966	0.966	471 (7.15)	969 (14.71)	1344 (1137-1554)
	C	0.956	0.946	0.953	0.951	510 (7.74)	699 (10.61)	3621 (2462-6025)
	D	0.876	0.968	0.764	0.798	195 (2.96)	407 (6.18)	737 (512-1074)
	E	0.846	0.967	0.707	0.747	41 (0.62)	67 (1.02)	686 (590-794)
**Race**								
	American Indian	0.9	1	0.8	0.833	<20 (<0.30)	<20 (<0.30)	1224 (858-1593)
	Asian^a^	0.917	1	0.833	0.862	<20 (<0.30)	30 (0.46)	1361 (1178-1705)
	Black	0.973	0.965	0.961	0.962	492 (7.47)	1173 (17.80)	1299 (1092-1581)
	Other^b^	0.989	1	0.979	0.983	47 (0.71)	32 (0.49)	1254 (1031-1478)
	Pacific Islander^c^	1	1	1	1	<20 (<0.30)	<20 (<0.30)	837 (612-1110)
	White	0.959	0.963	0.942	0.946	1858 (28.20)	2936 (44.56)	1339 (1027-1877)
**Age range (years)**								
	≤14	0.796	0.6	0.6	0.6	<20 (<0.30)	246 (3.73)	1603 (1218-2279)
	15-24	0.982	0.985	0.97	0.973	198 (3.01)	492 (7.47)	1183 (981-1523)
	25-34	0.964	0.971	0.958	0.961	692 (10.50)	682 (10.35)	1279 (1039-1693)
	35-44	0.954	0.957	0.957	0.957	805 (12.22)	717 (10.88)	1362 (1087-1823)
	45-54	0.956	0.962	0.934	0.94	458 (6.95)	753 (11.43)	1344 (1064-1748)
	55-64	0.951	0.98	0.907	0.921	216 (3.28)	679 (10.31)	1359 (1040-1761)
	≥65	0.882	0.871	0.771	0.789	35 (0.53)	611 (9.27)	1355 (1020-1796)
**Sex**								
	Female	0.959	0.961	0.943	0.947	736 (11.17)	1164 (17.67)	1381 (1081-1859)
	Male	0.964	0.965	0.947	0.951	1673 (25.39)	3016 (45.77)	1307 (1039-1711)
**Education**								
	8th grade or less	0.94	0.932	0.896	0.903	77 (1.17)	330 (5.01)	1485 (1153, 2156)
	9th-12th grade but no diploma	0.968	0.959	0.963	0.962	460 (6.98)	691 (10.49)	1304 (1035-1709)
	High school graduate or General Educational Development	0.961	0.967	0.944	0.948	1232 (18.70)	1808 (27.44)	1314 (1031-1726)
	Some college but no degree	0.965	0.969	0.948	0.953	368 (5.59)	591 (8.97)	1297 (1052-1664)
	Associate’s degree	0.946	0.935	0.926	0.928	108 (1.64)	209 (3.17)	1338 (1049-1745)
	Bachelor’s degree	0.969	0.977	0.944	0.950	89 (1.35)	309 (4.69)	1340 (1097-1700)
	Master’s degree	1	1	1	1	<20 (<0.30)	85 (1.29)	1324 (1119-1849)
	Doctorate or professional degree	0.989	0.75	1	0.938	<20 (<0.30)	44 (0.67)	1369 (1037-2200)
	Unknown	0.964	1	0.929	0.942	56 (0.85)	113 (1.71)	1507 (1207-2389)

^a^*Asian* includes Asian Indian, Chinese, Filipino, Korean, Vietnamese, and Other Asian.

^b^*Other* includes Other Race and Unknown.

^c^*Pacific Islander* includes Guamanian or Chamorro, Samoan, and Other Pacific Islander.

## Discussion

### Principal Findings

Narrative text was used to construct bag-of-words models (term frequency–inverse document frequency scoring) that predicted whether a given autopsy described a fatal overdose. The structure and details of the scene varied considerably across autopsies. Despite this heterogeneity in autopsy data, the SVM, random forest, and gradient boosted tree classifiers all showed excellent performance. Both SVM and random forest achieved the highest recall and *F*_2_-score, but only the random forest was calibrated.

The terms most strongly associated with fatal overdoses in both the random forest and gradient boosted tree models were aligned with known state-level trends. From 2015 to 2019, the rate of fatal drug overdoses involving fentanyl increased from 2.7 to 16.8 per 100,000 residents in Tennessee [25]. More than half of fentanyl overdoses involved additional substances in 2019. Unsurprisingly, “fentanyl,” “norfentanyl,” “4ANPP” (ie, “4-ANPP” before text preprocessing; a precursor to illicit fentanyl), and “combined” were among the top terms most strongly associated with fatal overdose autopsies. Overdoses have also been increasingly linked to methamphetamine and heroin, both of which are highly predictive of fatal overdoses [25]. This rise in fentanyl- and stimulant-related overdose deaths has also been observed at the national level [23,26].

Forensic center subgroup analysis revealed lower model discrimination on data from forensic centers D and E. These 2 forensic centers contributed the fewest autopsies. It is possible that the models were not given ample opportunities to learn from autopsies contributed by these 2 centers. Model performance may improve with the oversampling of autopsies from these 2 forensic centers. The subgroup analyses by race and age indicated lower *F*_2_-scores for the American Indian and Asian race groups as well as the ≤14 and ≥65 years age groups. These race and age subgroup results differed slightly in the logistic regression analysis, suggesting that the small sample sizes for these subgroups and others warrant additional study.

Subgroup differences in model performance and narrative length are worth additional study, particularly considering the findings of Mezuk et al [22]. In their study, Mezuk et al [22] identified shorter National Violent Death Reporting System narrative lengths for suicide and undetermined deaths among individuals who were male, were older, achieved lower education, and were part of certain racial and ethnic minority groups, compared with their counterparts. These findings suggest that models that use narrative text may generate biased predictions for individuals in certain subgroups. A similar detailed analysis is beyond the scope of this study. Our data did not suggest a noticeable difference in median narrative length across races. However, an analysis of narrative length using the Wilcoxon rank-sum test indicated a significant difference in narrative section length between the Black and White race subgroups (*P*<.001; Multimedia Appendix 4). These shorter sections did not appear to affect model performance, but further studies are needed.

Previous studies have used more complex machine learning methods, such as deep learning on autopsy text, to predict the cause of death ICD-10 codes [14,15,27]. Although such models may incorporate more information to perform generalized cause of death prediction, our models were specific to fatal overdose prediction. More specialized models may be better suited to identify fatal overdoses when the evidence is less clear-cut. For instance, drugs discovered at the death scene may not necessarily be linked to the primary cause of death. Alternatively, a fatal overdose may be the primary cause of death, but the death certificate may only reflect a contributing comorbidity. Our model performance metrics suggested that more advanced machine learning methods were not needed for this prediction task. As indicated by the relatively small size of the vocabulary set in this study (4002 terms across 10,215 autopsies) and subsequently verified by manual review, the language across autopsy reports was fairly standardized and uniform. Perhaps certain combinations of terms (eg, “fentanyl,” “accident,” and “toxicity”) were highly predictive of fatal overdose. Another study used NLP to predict fatal overdoses from free-text fields in Kentucky death certificates [13]. Similar to our study, bag-of-words–based machine learning models were used. However, whereas Kentucky relied on death certificate fields (as few as 2 words), our study used a different data source: autopsy narrative text. These sections ranged from hundreds to thousands of characters in length and provided additional contextual information in the form of scene evidence and medical history.

This work was completed in an academic-public partnership between the TDH and VUMC, where the SUDORS team at the TDH provided domain knowledge, and the Walsh Lab at VUMC provided modeling expertise. The goal was to develop a model that can be run and maintained by the SUDORS team. To this end, a simple modeling pipeline has several advantages. Adobe Acrobat Pro is easy to operate and does not require computer programming skills. Therefore, the OCR process can remain consistent, despite any changes in personnel that may occur. Sections of narrative text were identified using keywords that appeared as headings in autopsy reports. These rules can be modified to ensure the continued extraction of the appropriate autopsy sections, should autopsy templates change in the future. The bag-of-words models were agnostic to word order and less context dependent compared with more complex models such as word2vec. The simplicity of the bag-of-words approach translated into increased robustness to the variable quality of text extracted using OCR. Together, these aspects of the modeling pipeline facilitated the handoff of the code to the TDH SUDORS team and may inform modeling practices in other states.

### Limitations

This study has several limitations. The OCR results depended on the quality of the scanned autopsy reports. The OCR was less accurate when the reports were scanned crooked (as reported in the results, no more than 1% of all autopsies were removed for this reason). Sometimes, spaces would be missed (eg, “decedentwas” instead of “decedent was”), misspellings would be introduced, or the OCR would add symbols (often <4 characters long). These tendencies informed the decision to exclude terms appearing in fewer than 20 documents or those having fewer than 4 characters. Although the Adobe Acrobat Pro OCR was sufficient for this study, careful manual reviews should be conducted when applying this tool to different data sources. In addition, the text was extracted using a simple Python function that did not always maintain proper word order when the text spanned multiple columns. As word order was occasionally altered during the OCR text extraction process, the tested models did not consider negation. Although word-order–agnostic models were sufficient for the prediction task presented in this study, more precise text extraction methods may yield at least a modest improvement in recall. Other studies may assess the importance of considering negation in autopsy reports.

Fatal overdoses were identified by the TDH surveillance team through an automated process that involved ICD-10 codes and keyword searches of death certificates, followed by a manual review. Although this process has been improved and tested over the years, it depends on death certificate codes and cause of death text, both of which may be delayed. The NLP-based approach in this study has the potential to overcome this limitation, as it relies on autopsy narrative text (a proxy for medicolegal scene investigation documentation), which is often available much sooner and can provide more contextual information than death certificates. Furthermore, the Tennessee medicolegal death investigation system is decentralized, thus allowing a wide range of variability in the level of detail included in the autopsy narratives. Despite this variation, the content of the information conveyed remained consistent. Finally, this study involved only data from one state health department. Although the studied classifiers performed well, data from other states may require additional preprocessing steps and potentially model retraining.

### Future Work

The absence of a standardized autopsy template for all forensic centers added complexity to this prediction task and likely contributes to the additional time spent by the surveillance team in manually abstracting information. The recent adoption of the Medicolegal Death Investigation Log for some jurisdictions throughout the state is a step toward standardization, as this platform offers a common set of data fields for users to complete, in addition to a centralized location for documents to be uploaded. The utility of Medicolegal Death Investigation Log data in NLP models should be investigated in the future.

Efforts to standardize autopsy reports across forensic centers could facilitate the development of NLP tools specifically trained on text from autopsy reports. Such tools may identify scene evidence and naloxone administration more accurately than tools designed for electronic health record data, as described by Harris et al [28]. In the future, more specialized NLP tools could facilitate the automated extraction of public health–related variables for more complex NLP tasks.

Systematic characterization of the differential coverage of information related to scene evidence and medical history conveyed in autopsies from each forensic center may be worthwhile. This analysis may reveal factors contributing to poorer model performance and inform the standardization of statewide autopsy reports. As public health surveillance becomes more automated, it is increasingly important to consider variations conferred by nonstandardized documentation practices.

Given the findings of Mezuk et al [22] related to narrative lengths across different subgroups, future studies might seek to understand such patterns using data from all-cause deaths. Mezuk et al [22] also noted that missingness (ie, the presence or absence of a medical examiner narrative or law enforcement report) was associated with different social variables, such as homeless status, education, race, and ethnicity. These findings from Mezuk et al [22] are worth considering in future work because of the potential downstream effects of predictive models leveraging such biased data. In the case of public health surveillance, the result may be underreporting of deaths in certain groups, thus limiting intervention in communities that may be the most in need. This is particularly important, given the existing disparities exacerbated by the COVID-19 pandemic, which contributed to disproportionately high increases in overdose mortality rates among Black and American Indian or Alaska Native individuals compared with White individuals [29,30]. There is an urgent need to develop predictive models that ensure the appropriate allocation of resources during interventions.

Operationalizing the random forest classifier may facilitate rapid fatal overdose reporting in the future. OCR was performed using the batch processing option accessible through the Adobe Acrobat Pro user interface. The random forest classifier should be easy to scale to larger data sets and reduce overfitting by leveraging ensemble learning methods. Swift reporting can aid in the development of faster prevention and response activities at the community level, which can help curtail the growing number of fatal drug overdoses in Tennessee. Deploying this model could enhance the capacity of TDH to conduct fatal drug overdose surveillance.

### Conclusions

Narrative text from Tennessee autopsy reports was used to develop NLP models that predicted the likelihood that a given autopsy described a fatal overdose. Simple bag-of-words–based models were sufficient for identifying potential fatal overdoses for public health surveillance. Additional studies are needed to ensure that the random forest classifier facilitates timely fatal overdose reporting for individuals across all subgroups.

## Appendix

Multimedia Appendix 1 Manners of death among cases and controls, as indicated in death certificates.

Multimedia Appendix 2 Top 20 SHAP (Shapley additive explanations) values for gradient boosted tree classifier.

Multimedia Appendix 3 Logistic regression discrimination and classification metrics for each subgroup in the test set (n=6589).

Multimedia Appendix 4 Comparison of narrative section lengths by race subgroup in the entire data set (N=17,342).

## Abbreviations

AUROC: area under the receiver operating characteristic
ICD-10: International Classification of Diseases, Tenth Revision
NLP: natural language processing
OCR: optical character recognition
OSCME: Office of the State Chief Medical Examiner
REDCap: Research Electronic Data Capture
SHAP: Shapley additive explanations
SUDORS: State Unintentional Drug Overdose Reporting System
SVM: support vector machine
TDH: Tennessee Department of Health
VUMC: Vanderbilt University Medical Center

## Data Availability

The data sets analyzed in this study contain protected health information and are not publicly available; however, aggregate data may be available from the Office of Informatics and Analytics at the Tennessee Department of Health upon reasonable request [31].
