# Supplementary material for: Using Natural Language Processing to Predict Fatal Drug Overdose From Autopsy Narrative Text: Algorithm Development and Validation Study
Source: JMIR Public Health Surveill. 2023 May 19;9:e45246. doi: 10.2196/45246 (PMC10238956; doi:10.2196/45246)
Supplement: Multimedia Appendix 1 [file publichealth_v9i1e45246_app1.docx]

## Multimedia Appendix 1

Table 1. Manners of death among cases and controls, as indicated in death certificates.

|  | Cases | Controls |
| --- | --- | --- |
|  | N=5,912 | N=11,430 |
|  | n (%) | n (%) |
|  |  |  |
| Accidental | 5,811 (98) | 2,691 (24) |
| Natural | 5^a^ (0.08) | 3,994 (35) |
| Suicide | 0 (0) | 2,127 (19) |
| Homicide | 0 (0) | 2,017 (18) |
| Undetermined | 96 (1.6) | 601 (5) |

^a^Some cases may have been coded as *natural* if physicians sign the death certificate.
