# Supplementary material for: Using Natural Language Processing to Predict Fatal Drug Overdose From Autopsy Narrative Text: Algorithm Development and Validation Study
Source: JMIR Public Health Surveill. 2023 May 19;9:e45246. doi: 10.2196/45246 (PMC10238956; doi:10.2196/45246)
Supplement: Multimedia Appendix 2 [file publichealth_v9i1e45246_app2.docx]

## Multimedia Appendix 2


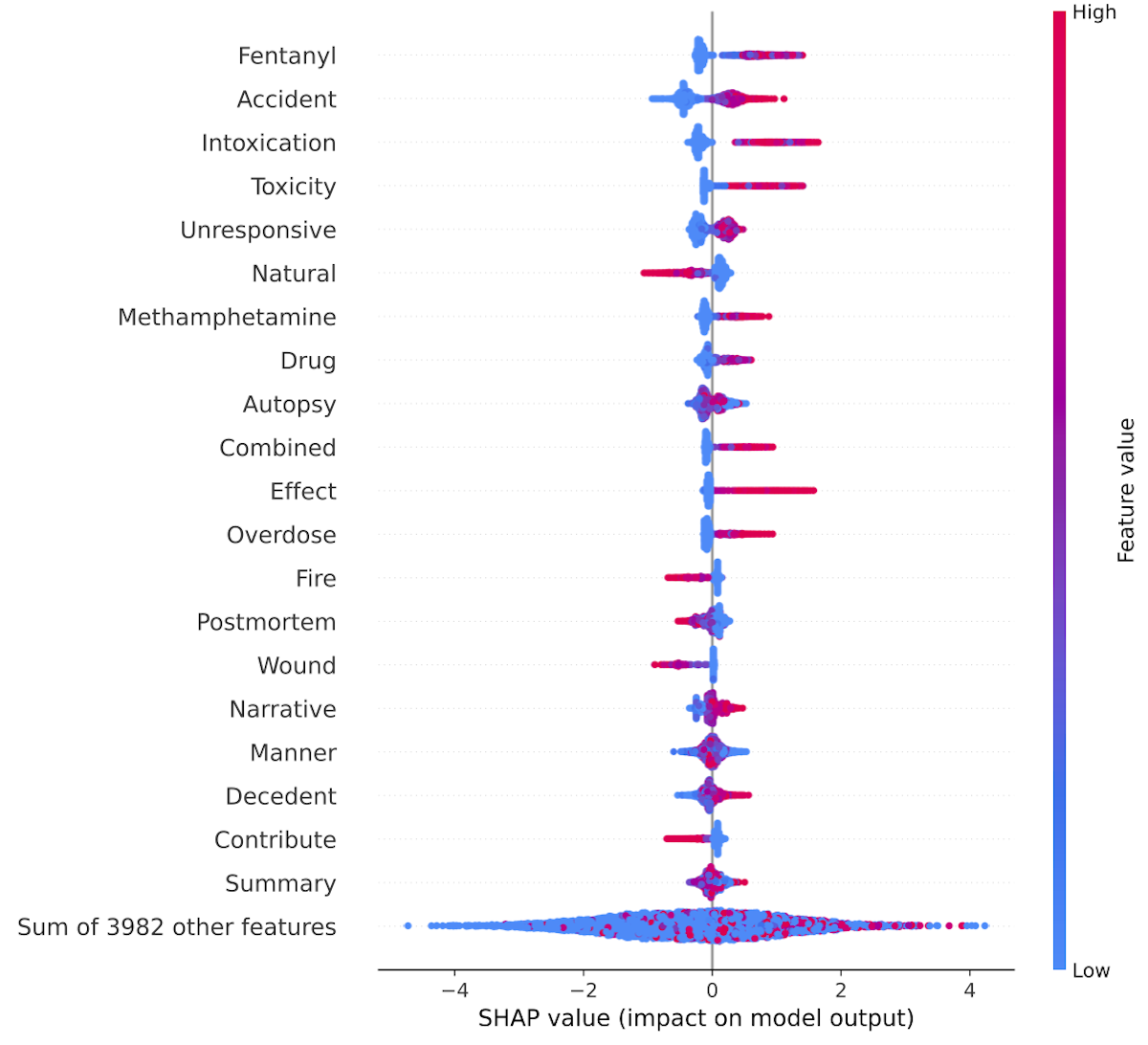
Figure 1. Top 20 Shapley Additive exPlanations (SHAP) values for the gradient boosted trees classifier.
