# Supplementary material for: Using Natural Language Processing to Predict Fatal Drug Overdose From Autopsy Narrative Text: Algorithm Development and Validation Study
Source: JMIR Public Health Surveill. 2023 May 19;9:e45246. doi: 10.2196/45246 (PMC10238956; doi:10.2196/45246)
Supplement: Multimedia Appendix 3 [file publichealth_v9i1e45246_app3.docx]

## Multimedia Appendix 3

Table 1. Logistic regression discrimination and classification metrics for each subgroup in the test set (N=6,589).

|  | Subgroup | AUROC | Precision | Recall | F_2_ |
| --- | --- | --- | --- | --- | --- |
|  |  |  |  |  |  |
| **Forensic Center** |  |  |  |  |  |
|  | A | 0.958 | 0.978 | 0.928 | 0.937 |
|  | B | 0.971 | 0.978 | 0.953 | 0.958 |
|  | C | 0.945 | 0.934 | 0.939 | 0.938 |
|  | D | 0.897 | 0.946 | 0.815 | 0.839 |
|  | E | 0.841 | 1 | 0.683 | 0.729 |
| **Race** |  |  |  |  |  |
|  | American Indian | 1 | 1 | 1 | 1 |
|  | Asian^a^ | 0.917 | 1 | 0.833 | 0.862 |
|  | Black | 0.963 | 0.973 | 0.937 | 0.944 |
|  | Other^b^ | 0.968 | 1 | 0.936 | 0.948 |
|  | Pacific Islander^c^ | 1 | 1 | 1 | 1 |
|  | White | 0.948 | 0.963 | 0.918 | 0.926 |
| **Age** |  |  |  |  |  |
|  | ≤14 | 0.598 | 0.5 | 0.2 | 0.227 |
|  | 15-24 | 0.977 | 0.984 | 0.96 | 0.964 |
|  | 25-34 | 0.956 | 0.973 | 0.939 | 0.946 |
|  | 35-44 | 0.945 | 0.952 | 0.943 | 0.945 |
|  | 45-54 | 0.939 | 0.965 | 0.897 | 0.91 |
|  | 55-64 | 0.928 | 0.995 | 0.856 | 0.881 |
|  | ≥65 | 0.856 | 0.926 | 0.714 | 0.749 |
| **Sex** |  |  |  |  |  |
|  | Female | 0.946 | 0.951 | 0.923 | 0.928 |
|  | Male | 0.954 | 0.973 | 0.922 | 0.931 |
| **Education** |  |  |  |  |  |
|  | 8^th^ grade or less | 0.926 | 0.971 | 0.857 | 0.878 |
|  | 9^th^-12^th^ grade but no diploma | 0.948 | 0.959 | 0.922 | 0.929 |
|  | High school graduate or  GED^d^ | 0.952 | 0.971 | 0.924 | 0.933 |
|  | Some college but no degree | 0.956 | 0.958 | 0.938 | 0.942 |
|  | Associate’s degree | 0.923 | 0.931 | 0.88 | 0.89 |
|  | Bachelor’s degree | 0.965 | 0.988 | 0.933 | 0.943 |
|  | Master’s degree | 0.969 | 1 | 0.938 | 0.949 |
|  | Doctorate or  professional degree | 0.989 | 0.75 | 1 | 0.938 |
|  | Unknown | 0.964 | 1 | 0.929 | 0.942 |

^a^*Asian* includes Asian Indian, Chinese, Filipino, Korean, Vietnamese, and Other Asian.

^b^*Other* includes Other Race and Unknown.

^c^*Pacific Islander* includes Guamanian or Chamorro, Samoan, and Other Pacific Islander.

^d^General Educational Development test
