# Supplementary material for: Using Natural Language Processing to Predict Fatal Drug Overdose From Autopsy Narrative Text: Algorithm Development and Validation Study
Source: JMIR Public Health Surveill. 2023 May 19;9:e45246. doi: 10.2196/45246 (PMC10238956; doi:10.2196/45246)
Supplement: Multimedia Appendix 4 [file publichealth_v9i1e45246_app4.docx]

## Multimedia Appendix 4

Table 1. Comparison of narrative section lengths by race subgroup in the entire dataset (N=17,342).

|  | Autopsy Reports | Narrative Length | Wilcoxon Rank-Sum^a^ |
| --- | --- | --- | --- |
|  | n | Median (IQR) | *P* value |
|  |  |  |  |
| American Indian | 22 | 1,410 (1,136-2,208) | 0.41 |
| Asian^b^ | 105 | 1,395 (1,148-1,821) | 0.13 |
| Black | 3702 | 1,283 (1,053-1,613) | <.001 |
| Other^c^ | 129 | 1,274 (1,072-1,652) | 0.51 |
| Pacific Islander^d^ | <20 | 1,110 (806-1,410) | 0.023 |
| White | 9883 | 1,346 (1,041-1,845) | --- |

^a^Two-tailed test, compared to White

^b^*Asian* includes Asian Indian, Chinese, Filipino, Korean, Vietnamese, and Other Asian.

^c^*Other* includes Other Race and Unknown.

^d^*Pacific Islander* includes Guamanian or Chamorro, Samoan, and Other Pacific Islander.
